# Absorption rate of krill oil and fish oil in blood and brain of rats

**DOI:** 10.1186/s12944-018-0812-7

**Published:** 2018-07-18

**Authors:** So Hyun Ahn, Su Jin Lim, Young Moo Ryu, Hye-Ryung Park, Hyung Joo Suh, Sung Hee Han

**Affiliations:** 1 Department of Food and Nutrition, Korea University, Seoul, 07249 Republic of Korea; 2 Alpha B&H, Seoul, 06705 Republic of Korea; 3 Department of Food Science and Biotechnology, Kyonggi University, Suwon, 16227 Republic of Korea; 4 BK21Plus, College of Health Science, Korea University, Seoul, 02841 Republic of Korea

**Keywords:** Krill oil, Phospholipids, Eicosapentaenoic acid, Docosahexaenoic acid, Bioavailability

## Abstract

**Background:**

Krill (*Euphausia superba*) is a small marine crustacean with a lipid content. The mechanism of Krill oil function is not clear yet and research reports on the absorption rate of the phospholipids of krill oil in the blood and brain are very poor.

**Methods:**

We studied the effect of oral short-term and long-term administration of Krill oils (KOs) on bioavailability in the blood and brain of rats. For short-term testing of fish and KO bioavailability, rats were divided into four groups: normal, fish oil (FO), Krill oil 1 (KO), and Krill oil 2 (CKO). The blood and brain were collected at 2, 4, 8, 12, 24, and 48 h after oral administration (1000 mg/rat). Five hundred milligrams of FO, KO, and CKO were orally administered daily for 2 weeks for long-term administration, and then the brain and blood were collected.

**Results:**

Two types of KOs showed high content of eicosapentaenoic acid (EPA) and docosahexaenoic acid (DHA) in the PL. The EPA content of CKO and KO were 41.13 and 32.49%, respectively. After short-term KO administration, KO showed a higher EPA content than CKO in the blood after 2 h. KO showed higher content of DHA than CKO even after 2 h. FO increased until 8 h, but then decreased rapidly until 12 h. Although the total unsaturated fatty acid (UFA) content of KOs was lower than the total UFS content in FO, the remaining UFS content in the brain was higher than that in FO over time. Following oral administration of FO, KO, and CKO for 1 and 2 weeks, triglycerides (TG) and PL contents in the blood for KOs were slightly higher than for FO. EPA and DHA levels in the brain were slightly higher in KOs following long-term administration, but the difference was not significant.

**Conclusions:**

Base on these findings, KOs have functional potential for the brain and vascular diseases, and can be utilized as a multi-functional material composed mainly of functional ingredients.

## Background

Unsaturated fatty acids (UFSs) have been reported to have beneficial effects on health [1]. Numerous studies have reported the health functions of fish oils (FOs) and their components such as docosahexaenoic acid (DHA) and eicosapentaenoic acid (EPA) [1, 2]. Polyunsaturated fatty acids (PUFAs) are important for maintaining healthy cells and hormone levels. N-6 fatty acids, primarily arachidonic acid, were shown to stimulate inflammatory processes by stimulating the flux of inflammatory pro-inflammatory type-2 prostaglandins and type-4 leukotrienes [3, 4]. The n-3 fatty acids, mostly EPA and DHA, modify cardiovascular and related diseases, but their mechanisms remain unclear [5, 6]. Increased intake of EPA and DHA may increase EPA and DHA in the tissues, cellular lipids, and circulatory lipids [7].

Krill (*Euphausia superba*) is a small marine crustacean with a lipid content of 12–50%. KOs contain astaxanthin, an antioxidant molecule, and high concentrations of n-3 unsaturated fatty acids (n-3 LCPUFA) (30–65%) [8, 9]. Researches have shown that n-3 LCPUFAs in PLS form are more bioavailable and more efficiently absorbed, particularly by brain tissue [10, 11]. Because of the large amounts of astaxanthin and n-3 LCPUFAs in KOs, these oils have positive effects on cardiovascular disease (plasma triglyceride, platelet aggregation, and inflammatory marker reduction) [12], insulin resistance, and neurocognition [13].

KOs are abundant in n-3 fatty acids, phospholipids, and various antioxidants that are generally different from those in FOs [14, 15]. Fatty acids in FO mainly include triglycerides, while in KOs, 30–65% of fatty acids are present as phospholipids, the main component of the cell membrane [16]. Binding between phospholipids and n-3 fatty acids greatly promotes the passage of molecules through the intestinal wall, increasing absorption rates and ultimately improving n-3: n-6 fatty acid ratios [17, 18]. These KOs not only confer various functions, but also have high bioavailability, so small amounts of KOs still exhibit positive functions. The demand for functional materials or functional foods for treating various geriatric diseases that occur with aging is currently increasing. Particularly, it is urgent to search for and develop functional materials for senile dementia such as Alzheimer’s disease. Studies of phosphatidylserine having n-3 PUFAs revealed improvement in cerebellum and cerebral cortex function in the elderly [19, 20]. However, studies of the bioavailability of KOs are limited, particularly the bioavailability of KOs in the blood and brain.

We investigated the effects of oral short-term and long-term administration of KOs on bioavailability in the blood and brain of rats. The results of this study suggest that Kos are effective functional materials in the brain.

## Methods

### Animals and oils

Male Sprague-Dawley rats (150–200 g, 6 weeks old) were purchased from Orient Bio (Seoul, Korea) and grown in individual cages. Experimental animals were kept at a temperature of 21 ± 1 °C and relative humidity of 50–55%. Water and feed were provided ad libitum during the adaptation period of 1 week. All experiments were approved by the Ethics Committee for Use of Experimental Animals at Kyonggi University (2017–006). FO and KOs were prepared by Apha B&H Co. Ltd. (Chungcheongbuk-do, Korea). FO contained DHA (324.00 mg/g), EPA (484.00 mg/g), and n-3 (886.00 mg/g). KOs contained DHA (76.24 mg/g), EPA (116.99 mg/g), phospholipid (263.00 mg/g), phosphatidylcholine in phospholipid (990.01 mg/g of phospholipid), and astaxanthin (0.19 mg/g). All chemicals were reagent-grade.

### Short-term and long-term administration

For short-term evaluation of fish and KO bioavailability, rats were divided into 4 groups (6 rats/group): normal, FO (FO), KO 1 (KO), and KO 2 (CKO). KO1 is a product made by enzyme extraction and CKO is a product made by solvent extraction. After 24 h starvation, saline, FO, and KOs were orally administered (1000 mg/rat). The blood and brain were collected after 2, 4, 8, 12, 24, 48 h of oral administration. The groups for the long-term tests were divided in the same manner as for the short-term test. Saline, FO, KO, and CKO (500 mg) were orally administered every day to each rat for 2 weeks. After 2 weeks, rats sacrificed, and blood and brains were collected.

### Triglyceride and phospholipid of oils

Triglyceride (TG) and phospholipid analysis (PL) was conducted by thin-layer chromatography (TLC) [21]. To detect TG and PL, 30 mg of sample dissolved in chloroform was placed on a TLC silica gel plate (Merck KGaA, Darmstadt, Germany) and 100 μL of samples were loaded. The development solvent contained chloroform, methanol, acetic acid, water (75: 40: 8: 3). After loading, the TLC plate was dried, and then 0.2% 2,7-dichlorofluorescein reagent (in 95% methanol) was sprayed onto the TLC plate. TG and PL were identified using a UV detector. Bands corresponding to TG and PL were scraped off and methylated with 14% BF_3_ in methanol. The resulting fatty acid methyl ester was analyzed by gas chromatography [22].

### TG in plasma

Blood from the portal vein was collected in a heparinized microcentrifuge tube (containing 20 μL of 1000 IU heparin/mL of blood). Plasma separated by centrifugation was analyzed using the FUJI DRI CHEM 3500 (Tokyo, Japan).

### Lipid extraction of plasma and brain

Lipid extraction for fatty acid analysis was performed as described by Folch with some modifications [23]. Lipid in 1 mL of plasma and brain were extracted in 4 mL of chloroform: methanol = 2:1 mixture. Ten micrograms of heptadecanoic acid were added as an internal standard. The supernatant was removed by nitrogen purging. After acid hydrolysis using 8.3 M HCl, methylation was carried out and the hexane layer was analyzed by gas chromatography.

### Gas chromatography analysis

A gas chromatograph (Varian 3800; Varian Inc., Walnut Creek, CA, USA) fitted with a Supelcowax 10 fused-silica capillary column (30 m length, 0.25 mm id., 0.25 μm film thickness; Supelco, Bellefonte, PA, USA) and flame ionization detector were used [24]. Initially, the column was maintained at 180 °C for 1 min and then increased to 230 °C at a rate of 1.5 °C/min. The temperature was then held at 230 °C for 10 min. Helium was used as a carrier gas at a flow rate of 1 mL/min and a split ratio of 50:1. The injector and detector temperatures were 240 °C and 250 °C, respectively. Fatty acid methyl esters were verified against the retention times of known standards.

## Results

### TG and PLs in KOs

**Fig. 1:**
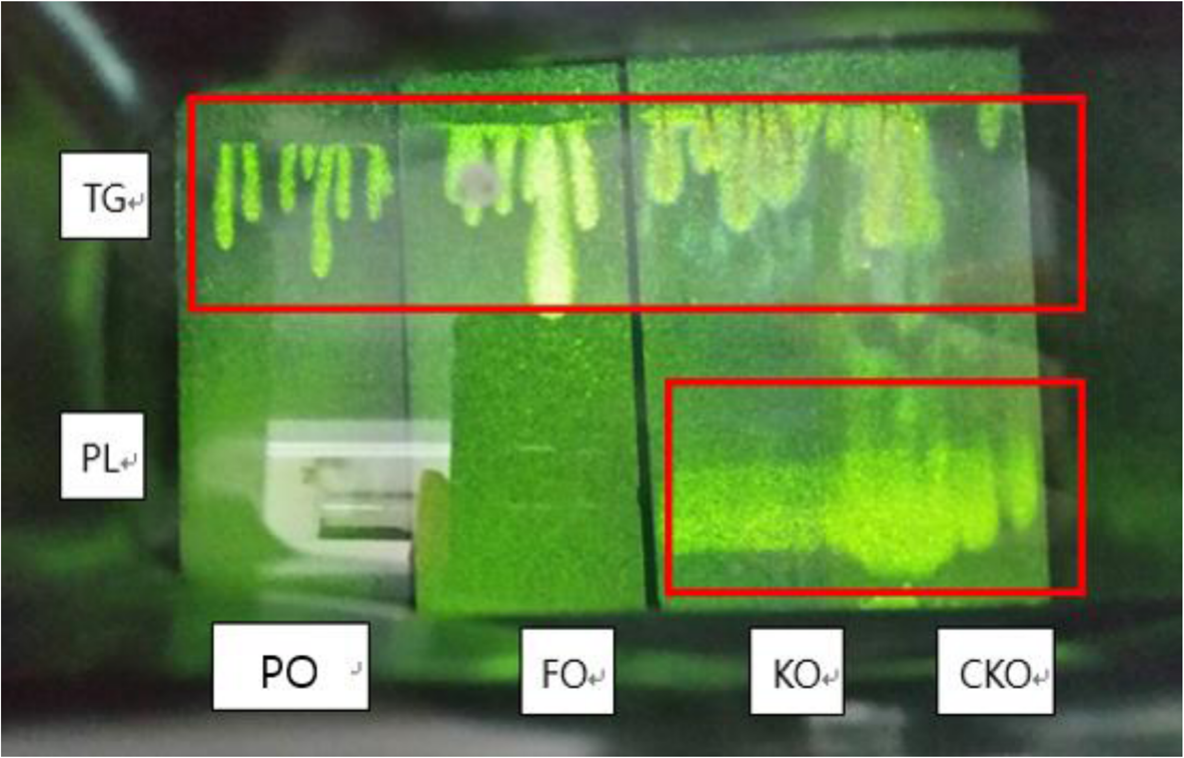
Separation of triglyceride (TG) and phospholipid (PL) in fish oil and krill oils using thin-layer chromatography. Perilla oil (PO) was as a control and sample oils were fish oil (FO), krill oil 1 (KO), and krill oil 2 (CKO)

To investigate the bioavailability of KOs, we analyzed TG and PLs in KOs by TLC (Fig. 1). Perilla oil and FO were used as standard samples. In addition, the fatty acid compositions of TG and PLs were analyzed (Tables 1 and 2). Because KOs have a high PL content, they were compared with perilla and FOs. PL was not detected in FO and perilla oil, but was detected in both types of KOs. TG was detected in all oils, but was most abundant in FO (Fig. 1). These results reveal the differences between FO and KOs. Tables 1 and 2 show the results of fatty acid analysis after oil was eluted by scraping TG and PL spots separated from FO and KOs. For the fatty acid composition of TG in fish and KOs, myristic acid (14:0) and palmitic acid (16:0), which are animal fatty were, showed significantly higher in KOs than FO (Table 1). In addition, UFSs such as oleic acid (18:1, n-9), vaccenic acid (18:1, n-7), linoleic acid (18:2, n-6), gamma linoleic acid (18:3, n-3), stearidonic acid (18:4, n-3), and gadoleic acid (20:1, n-9) were significantly higher in KOs than FO. In contrast, the content of EPA and DHA in FO was more than 2-fold higher. Mysteric acid and palmitic acid were 15.13 and 23.47% in KOs, respectively, which are similar to the values reported by Gigliotti et al. [25]. In contrast, palmitoleic acid and oleic acid were lower in our KOs than the contents described previously. This may be because of differences in the treatment with oil [25]. Table 2 shows the fatty acid composition in PL in FO and KOs. FO contained no PL, but the fatty acid composition in the PL of KOs was found. Particularly, two KOs showed high contents of EPA and DHA in PL. The EPA content of CKO was 41.13% and EPA content of KO was 32.49%. The EPA content of CKO was significantly higher than that of KO. However, the content of DHA was 19.76% for KO and 15.81% for CKO, indicating that the content in KO was significantly higher.

**Table 1:** Fatty acid composition in triglyceride (TG) of fish oil and kill oils

TG (Area %)	FO^1)^	KO^2)^	CKO^3)^
14:0	0.03 ± ^4)^ 0.00^b^	15.13 ± 0.09^a^	16.67 ± 0.10^a^
16:0	0.09 ± 0.00^c^	23.46 ± 0.02^a^	17.39 ± 0.04^b^
16:1	0.10 ± 0.00^c^	5.73 ± 0.04^b^	7.61 ± 0.05^a^
18:0	0.12 ± 0.00^c^	1.13 ± 0.01^a^	0.81 ± 0.01^b^
18:1(n-9)	0.25 ± 0.00^c^	11.70 ± 0.02^a^	10.90 ± 0.01^ab^
18:1(n-7)	0.09 ± 0.00^b^	7.97 ± 0.08^a^	7.51 ± 0.17^a^
18:2(n-6)	0.15 ± 0.00^b^	2.33 ± 0.04^a^	2.24 ± 0.01^a^
18:3(n-3)	0.06 ± 0.00^b^	0.12 ± 0.01^a^	0.00 ± 0.00^c^
18:4(n-3)	0.07 ± 0.00^c^	1.04 ± 0.02^a^	0.84 ± 0.04^b^
20:1(n-9)	0.11 ± 0.02^a^	0.06 ± 0.03^a^	0.00 ± 0.00^b^
20:2	0.19 ± 0.01^a^	0.06 ± 0.03^b^	0.00 ± 0.00^c^
20:4(n-6)	3.01 ± 0.01^a^	0.35 ± 0.02^c^	0.51 ± 0.00^b^
20:5(EPA)	57.71 ± 0.04^a^	19.92 ± 0.07^b^	22.83 ± 0.13^b^
22:5(DPA)	2.82 ± 0.01^a^	0.43 ± 0.01^b^	0.36 ± 0.02^b^
22:6(DHA)	35.21 ± 0.04^a^	10.60 ± 0.02^c^	12.33 ± 0.07^b^
Total	100	100	100

^1)^FO: Fish oil, ^2)^KO: Krill oil 1, ^3)^CKO: Krill oil 2, ^4)^ ± Mean standard deviation, One-way analysis of variance with Duncan’s multiple comparison test with mean values

**Table 2:** Fatty acid composition in phospholipid (PL) of fish oil and krill oils

PL (Area %)	FO^1)^	KO^2)^	CKO^3)^
14:0	Not detected	2.22 ± ^4)^0.04^b^	3.46 ± 0.02^a^
16:0		28.10 ± 0.30^a^	26.12 ± 0.08^a^
16:1		0.97 ± 0.03^b^	1.49 ± 0.02^a^
18:0		1.03 ± 0.02^a^	0.57 ± 0.01^b^
18:1(n-9)		5.39 ± 0.08^a^	4.26 ± 0.01^b^
18:1(n-7)		5.43 ± 0.14^a^	4.20 ± 0.03^b^
18:2(n-6)		1.52 ± 0.02^b^	1.72 ± 0.02^a^
18:3(n-3)		1.42 ± 0.00^a^	0.00 ± 0.00^b^
18:4(n-3)		0.50 ± 0.01^a^	0.23 ± 0.02^b^
20:1(n-9)		0.00 ± 0.00^a^	0.00 ± 0.00^a^
20:2		0.00 ± 0.00^a^	0.00 ± 0.00^a^
20:4(n-6)		0.26 ± 0.01^b^	0.40 ± 0.03^a^
20:5(EPA)		32.49 ± 0.46^b^	41.13 ± 0.02^a^
22:5(DPA)		0.91 ± 0.04^a^	0.62 ± 0.02^b^
22:6(DHA)		19.76 ± 0.35^a^	15.81 ± 0.05^b^
Total		100	100

^1)^FO: Fish oil, ^2)^KO: Krill oil 1, ^3)^CKO: Krill oil 2, ^4)^ ± Mean standard deviation, One-way analysis of variance with Duncan’s multiple comparison test with mean values

### Composition of SFA, monounsaturated fatty acid (MUFA), and PUFA

We also investigated the composition of saturated fatty acid (SFA), MUFA, and PUFA in TG and PL of the oils (Table 3). SFA and MUFA in TG of FO were significantly lower than in KO and CKO. In contrast, PUFA in TG of FO was the highest at 99.22%. Fatty acids in PL of FO was not detected, but fatty acids in PL of KO and CKO was detected. KO showed a higher MUFA content than CKO, and CKO showed a higher PUFA content.

**Table 3:** Composition ratio of saturated fatty acid (SFA), monounsaturated fatty acid (MUFA), and polyunsaturated fatty acid (PUFA) in fish oil and krill oils

Area %		SFA	MUFA	PUFA
TG^1)^	FO	0.24 ± 0.00^c^	0.55 ± 0.03^c^	99.22 ± 0.13^a^
	KO	39.71 ± 0.11^a^	25.46 ± 0.17^a^	34.84 ± 0.21^c^
	CKO	34.88 ± 0.15^b^	26.02 ± 0.23^a^	39.10 ± 0.28^b^
PL^2)^	FO	Not detected		
	KO	31.35 ± 0.36^a^	11.79 ± 0.26^a^	56.86 ± 2.32^b^
	CKO	30.15 ± 0.11^a^	9.94 ± 0.06^b^	59.91 ± 0.16^a^

^1)^TG: triglyceride, ^2)^ PL: phospholipids, SFA: 14:0, 16:0, 18:0; MUFA: 16:1, 18:1(n-9), 18:1(n-7); PUFA: 18:2(n-6), 18:3(n-3), 18:4(n-3), 20:4(n-6), 20:5, 22:5, 22:6. Values are presented as the mean ± S.E (n = 4). One-way analysis of variance with Duncan’s multiple comparison test with mean values

### Effect of KOs short-term administration on fatty acid composition in blood

**Fig. 2:**
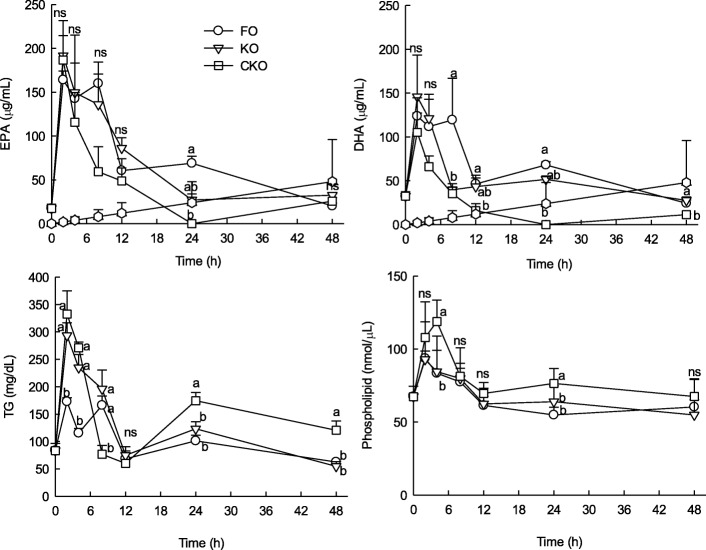
Changes of eicosapentaenoic acid (EPA), docosahexaenoic acid (DHA), TG (triglycerides), and phospholipid in plasma after short-term administration of fish oil (FO), krill oil 1 (KO), and krill oil 2 (CKO)

Figure 2 shows the contents of EPA, DHA, TG, and PL in the blood after a 2, 4, 8, 12, and 24 h of oral administration of KO, FO, and CKO. Regardless of the oil type, EPA and DHA tended to increase until 2 h after oral administration. KO showed a higher EPA content than CKO after 2 h. Particularly, KO showed higher contents of DHA than CKO even after 2 h. FO generally increased until 8 h, but decreased rapidly until 12 h.

The amount of TG in the blood was highest in CKO until 4 h, but there was no significant difference from KO. After 2 h of oral administration of CKO and KO, the TG value decreased. For CKO, the amount of TG in the blood was higher than that in the other groups at 24 and 48 h. The TG absorption rate of FO was not high but was maintained at a similar level and then decreased.

### Effect of KOs short-term administration on fatty acid composition in the brain

**Fig. 3:**
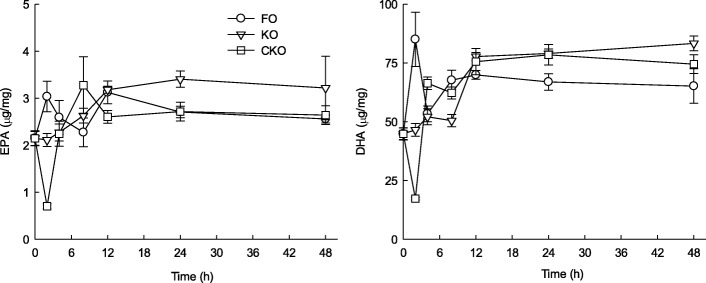
Changes in eicosapentaenoic acid (EPA) and docosahexaenoic acid (DHA) in brain lipid after short-term administration of fish oil (FO), krill oil 1 (KO), and krill oil 2 (CKO)

The results of analysis of brain EPA and DHA after 2, 4, 8, 12, 24, and 48 h are shown in Fig. 3. EPA and DHA contents gradually increased over time, whereas FO showed the highest level at 2 h and decreased slowly after oral administration of KO and CKO. Regardless of the type of oil, MUFA and PUFA increased over time (Table 4). Notably, although the total UFS content of the KOs was lower than the total UFS content of the FO, the remaining UFS content was higher than that of the FO over time. Studies of the relative oral bioavailability of n-3 supplements have shown controversial results, and several studies reported that phospholipid form (krill) is better absorbed than ethyl ester or TG type FO [16, 26].

**Table 4:** Composition of SFA, MUFA, and PUFA in brain after oral administration of fish oil and krill oils

mg/g brain		SFA	MUFA	PUFA
0		134.23 ± 6.17	62.49 ± 3.46	92.95 ± 5.10
2	FO	157.08 ± 30.48	79.37 ± 10.49	145.54 ± 20.01
	KO	137.95 ± 6.38	64.00 ± 3.50	95.25 ± 5.38
	CKO	52.42 ± 1.70	23.97 ± 0.57	35.88 ± 1.56
4	FO	160.02 ± 11.26	69.70 ± 2.47	109.89 ± 7.97
	KO	149.65 ± 10.97	71.58 ± 7.64	105.48 ± 7.23
	CKO	187.23 ± 9.45	89.63 ± 4.26	135.81 ± 5.74
8	FO	156.47 ± 5.70	70.65 ± 2.13	120.94 ± 7.10
	KO	145.98 ± 7.48	68.09 ± 4.72	107.60 ± 9.62
	CKO	174.99 ± 7.58	83.34 ± 3.59	128.49 ± 6.97
12	FO	209.72 ± 6.57	96.30 ± 2.48	144.63 ± 5.07
	KO	231.62 ± 6.25	106.51 ± 2.38	159.17 ± 3.91
	CKO	208.54 ± 9.68	98.26 ± 4.91	152.75 ± 10.79
24	FO	201.37 ± 10.29	91.27 ± 4.10	135.75 ± 7.05
	KO	233.60 ± 6.30	109.92 ± 3.66	162.32 ± 4.47
	CKO	220.05 ± 9.62	103.52 ± 5.41	158.58 ± 9.19
48	FO	200.00 ± 18.25	86.65 ± 8.88	130.62 ± 13.96
	KO	249.91 ± 10.49	114.06 ± 3.53	171.97 ± 6.39
	CKO	211.17 ± 8.76	98.65 ± 3.22	150.26 ± 7.70

SFA: 14:0, 16:0, 18:0; MUFA: 16:1, 18:1(n-9), 18:1(n-7); PUFA: 18:2(n-6), 18:3(n-3), 18:4(n-3), 20:4(n-6), 20:5, 22:5, 22:6. Values are presented as the mean ± S.E (*n* = 4). One-way analysis of variance with Duncan’s multiple comparison test with mean values

### Effect of KOs long-term administration on fatty acid composition in blood and brain

**Fig. 4:**
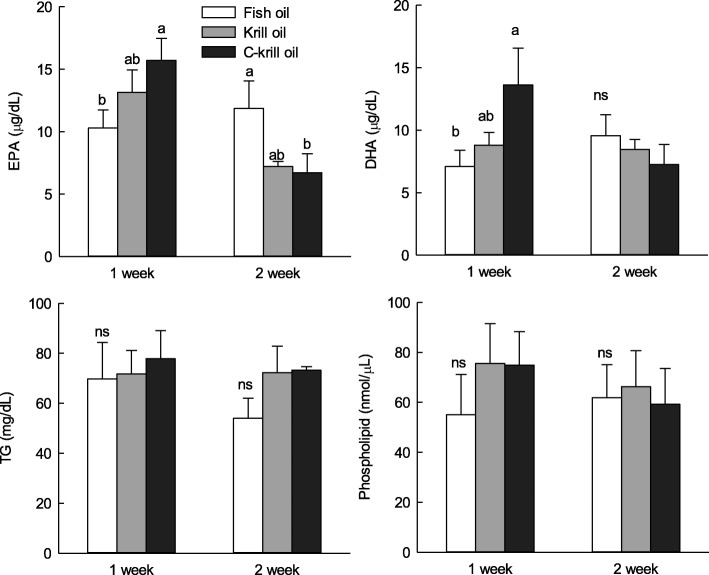
Changes in eicosapentaenoic acid (EPA), docosahexaenoic acid (DHA), TG (triglycerides), and phospholipid in plasma after long-term administration of fish oil (FO), krill oil 1 (KO), and krill oil 2 (CKO)

**Fig. 5:**
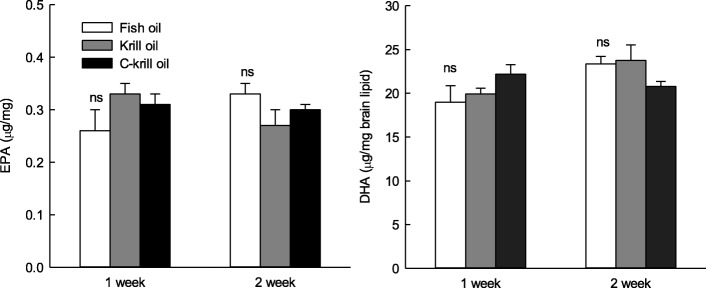
Changes in eicosapentaenoic acid (EPA) and docosahexaenoic acid (DHA) in brain lipid after long-term administration of fish oil (FO), krill oil 1 (KO), and krill oil 2 (CKO)

Changes in EPA and DHA contents during 2 weeks of oral administration of oils are shown in Fig. 4. The contents of EPA and DHA in the blood of FO, KO, and CKO were highest in CKO (15.71 and 13.75 μg/mg) at 1 week. FO showed higher EPA contents (11.86 μg/mg) than KO and CKO after 2 weeks. There was no significant difference in DHA content between FO, KO, and CKO after oral administration for 2 weeks. The changes in TG and PL contents in the blood after oral administration of FO, KO, and CKO for 1 and 2 weeks showed that KOs were slightly higher than FO, but the difference was not significant. These tendencies were similar in the brain (Fig. 5). CKO decreased gradually as the period of administration increased, whereas the content of FO and KO was initially low, but the absorption rate was higher following continuous consumption. However, there were no significant differences between groups. The amount of EPA and DHA in the brain was slightly higher in KOs than in the long-term administration period, but there was no significant difference. The results are also shown in Table 5.

**Table 5:** Composition of SFA, MUFA, and PUFA in the brain after long-term administration of fish oil and krill oils

	SFA	MUFA	PUFA
1 week (μg/g brain)			
FO	51.49 ± 4.64	26.45 ± 2.58	36.81 ± 3.66
KO	57.67 ± 1.94	28.28 ± 0.90	39.56 ± 1.34
CKO	60.21 ± 3.33	31.34 ± 1.93	43.47 ± 2.25
2 weeks (μg/g brain)			
FO	61.34 ± 2.26	32.85 ± 1.35	44.84 ± 1.88
KO	59.67 ± 3.30	32.36 ± 2.05	46.00 ± 3.48
CKO	46.00 ± 3.48	30.09 ± 0.97	40.58 ± 1.19

SFA: 14:0, 16:0, 18:0; MUFA: 16:1, 18:1(n-9), 18:1(n-7); PUFA: 18:2(n-6), 18:3(n-3), 18:4(n-3), 20:4(n-6), 20:5, 22:5, 22:6. Values are presented as the mean ± S.E (n = 4). One-way analysis of variance with Duncan’s multiple comparison test with mean values

## Discussion

Our results of TG and PLs in oils are similar to those reported in other studies; because PL and TG require different digestive enzymes, the bioavailability and tissue attachment of n-3 PUFA may differ. In turn, this can lead to other physiological and health effects [27]. Thus, EPA and DHA esterified with PLA in KOs may greatly impact human health. However, it is unusual to preferentially esterify EPA and DHA with PL [28]. The composition of saturated fatty acid (SFA), MUFA, and PUFA in TG and PL of the oils (Table 3) were investigated. Fatty acids were not detected in PL of FO but fatty acids were detected in PL of KO and CKO. KO showed higher MUFA content than CKO, and CKO showed higher PUFA content. These results indicate that the bioavailability of UFA, which is relatively high in TG of FO, and UFA, and are abundant in PL of KOs. In result of short-term administration on blood fatty acid composition, the absorption rate of PL in the blood was highest in CKO. FO showed a similar tendency to KO but had the lowest value. The absorption rate of n-3 fatty acids is affected by the chemical form and an excellent absorption rate for PL-bound n-3 fatty acids in KOs has been suggested [15, 16, 26, 29]. Considering that PL was not detected in the FO sample, our results are reasonable. EPA and DHA contents gradually increased over time, whereas FO showed the highest level at 2 h and decreased slowly after oral administration of KO and CKO in brain. Regardless of the type of oil, MUFA and PUFA increased over time (Table 4). DHA is particularly concentrated in the brain, nervous tissue, and retina and is essential for normal neurological function. Deficiency of DHA is associated with many neurological disorders DHA absorption is greater when it is delivered by liposomes than by oil. This was demonstrated by an increase in the DHA ratio in both lymphatic triacylglycerol and PL delivered by liposomes compared to by the FO diet [17]. KO is a complex combination of multiple active ingredients with synergistic bio- activities.

The brain exclusively consumes DHA in the form of lysophosphatidylcholine (LPC). Recent studies showed that in the blood brain barrier (Mfsd2a), the carrier transports LPC-DHA, but not DHA. Therefore, it is necessary to increase the level of LPC-DHA in the plasma to efficiently concentrate DHA in the brain [30, 31]. The exact mechanism of KOs is not clear. However, the unique biomolecular profile of KOs has demonstrates the possibility of erosion of n-3 (EPA/DHA) fatty acids in PLs and distinguishes KOs from FO. Also, the contents of EPA and DHA in the blood of FO, KO, and CKO were highest in CKO at 1 week. FO showed higher EPA contents than KO and CKO after 2 weeks. There was no significant difference in DHA content between FO, KO, and CKO after oral administration for 2 weeks. The changes in TG and PL contents in the blood after oral administration of FO, KO, and CKO for 1 and 2 weeks showed that KOs were slightly higher than FO, but the difference was not significant. These tendencies were similar in the brain.

FO is a better source for n-3 PUFA than KO. However, the bioavailability of n-3 PUFAs from krill oil (mainly PL) is as, or possibly more, efficient as n-3 PUFA from fish oil (TG). This supports the results of a study with krill oil and menhaden oil in humans [32]. In addition, the potential function of KO suggests that bioavailability to certain phospholipid-rich regions, such as the brain in the body, may be higher than FO.

## Conclusion

By evaluating the absorption rate of KOs following short-term and long-term administration, the contents of EPA and DHA remaining in the blood and brain after KOs ingestion were higher than those following ingestion of FO. This may be because of the chemical structure of the unique lipid component (phospholipid) in KOs. Therefore, KOs have functional potential for the brain and vascular diseases, and can be utilized as a multi-functional material composed mainly of functional ingredients.

## Abbreviations

DHA: Docosahexaenoic acid
EPA: Eicosapentaenoic acid
FO: Fish oil
KOs: Krill oils
PL: Phospholipid analysis
PUFAs: Polyunsaturated fatty acids
TG: Triglyceride
TLC: Thin-layer chromatography
UFSs: Unsaturated fatty acids
